# Avian extraembryonic membranes respond to yolk corticosterone early in development

**DOI:** 10.1242/bio.060131

**Published:** 2024-01-22

**Authors:** Emily P. Harders, Mitch Agustin, Ryan T. Paitz

**Affiliations:** Illinois State University, School of Biological Sciences, Campus Box 4120, Normal, IL 61790, USA

**Keywords:** Hormone-mediated maternal effects, Extraembryonic membranes, Glucocorticoid, Metabolism, Chicken

## Abstract

During times of maternal stress, developing embryos can be exposed to elevated levels of glucocorticoids, which can affect development and permanently alter offspring phenotype. In placental species, the placenta mediates fetal exposure to maternal glucocorticoids via metabolism, yet the placenta itself responds to glucocorticoids to regulate offspring growth and development. In oviparous species, maternal glucocorticoids can be deposited into the egg yolk and are metabolized early in development. This metabolism is mediated by the extraembryonic membranes, but it is unknown if the extraembryonic membranes also respond to maternal glucocorticoids in a way comparable to the placenta. In this study, we quantified the expression of acyl-CoA thioesterase 13 (*Acot13*) as an initial marker of the membrane's response to corticosterone in chicken (*Gallus gallus*) eggs. *Acot13* regulates fatty acid processing in the embryo, to potentially regulate resource availability during development. We addressed the following questions using *Acot13* expression: 1) Do the extraembryonic membranes respond to yolk corticosterone early in development? 2) Is the response to corticosterone dependent on the dose of corticosterone? 3) What is the duration of the response to corticosterone? 4) Does a metabolite of corticosterone (5β-corticosterone) elicit the same response as corticosterone? We found that corticosterone significantly induces the expression of *Acot13* on day four of development and that expression of *Acot13* increases with the dose of corticosterone. Further, we found expression of *Acot13* is significantly elevated by corticosterone on days four and six of development compared to oil treated eggs, but not on days eight and ten. Although this response is transient, it occurs during a critical period of development and could initiate a cascade of events that ultimately alter offspring phenotype. Finally, we found that 5β-corticosterone does not increase the expression of *Acot13*, indicating that metabolism inactivates corticosterone. Ultimately, this study provides insight into the mechanisms underlying how maternally deposited glucocorticoids can affect embryonic development.

## Teaser

**Summary:** Steroid exposure during embryonic development influences a variety of processes and here we show that the developing extraembryonic membranes respond to corticosterone early in development.

## INTRODUCTION

### Maternal stress effects are mediated by glucocorticoids

When vertebrates experience stress, glucocorticoids are produced via activation of the hypothalamic-pituitary-adrenal (HPA) axis to modulate and regulate the physiologic stress response (Sapolsky et al., 2000). Glucocorticoids regulate essential processes such as energy availability, via mobilizing existing energy stores and inhibiting the storage of available energy, while simultaneously suppressing non-essential processes such as reproduction. When female vertebrates experience stress during times of reproduction (pregnancy or egg production), offspring development can be affected. These ‘maternal stress effects’ are often caused by developing embryos being exposed to elevated glucocorticoids at sensitive periods of development. In placental vertebrates, elevated glucocorticoids in a pregnant female during reproduction can result in pregnancy loss (Nepomnaschy et al., 2006), decreased birth weight, and altered activity of the offspring's HPA axis (Mulder et al., 2002; Moisiadis and Matthews, 2014; Weinstock, 2008). In egg-laying vertebrates, glucocorticoids can be deposited into the egg yolk if maternal stress coincides with egg production (Almasi et al., 2012). This transfer of glucocorticoids has been observed in many avian species such as barn swallows (Saino et al., 2005), house wrens (Bowers et al., 2016; Heppner et al., 2023), Japanese quail (Hayward and Wingfield, 2004), and chickens (Rettenbacher et al., 2005). These maternally deposited glucocorticoids can permanently alter offspring physiology and behavior by acting during critical points in development (Henriksen et al., 2011). Effects include decreased hatch mass and altered activity of the HPA axis (Hayward and Wingfield, 2004, reviewed in Henriksen et al., 2011). Given the prevalence of effects elicited by embryonic glucocorticoid exposure in both placental and egg-laying vertebrates, there is a strong interest in understanding how embryonic glucocorticoid exposure is regulated.

### Extraembryonic tissues metabolize glucocorticoids

The degree of embryonic exposure to maternal glucocorticoids can vary with reproductive method. In placental species, where maternal and fetal physiology are connected for the entirety of gestation, the placenta regulates embryonic exposure to maternal glucocorticoids via metabolism (Seckl and Holmes, 2007). Maternal glucocorticoids such as cortisol are metabolized in the placenta via expression of 11β-hydroxysteroid dehydrogenase 2 (11β-HSD2), which converts active cortisol into inactive cortisone (Blasco et al., 1986). In oviparous species, despite the embryo developing separately from maternal circulation, regulation of embryonic exposure to maternally deposited glucocorticoids is mediated by the extraembryonic membranes (McNatt et al., 1992; von Engelhardt et al., 2009; Vassallo et al., 2014, 2019). In chicken eggs, extraembryonic membranes metabolize yolk corticosterone into two primary metabolites: 5β-corticosterone and 20β-corticosterone before day four of development (Harders et al., 2023). The necessary enzymatic transcripts, for 5β-reductase (*AKR1D1*) and 20β-hydroxysteroid dehydrogenase (*CBR1*), are transcribed in the membranes. These findings demonstrate that the extraembryonic membranes are capable of metabolically inactivating maternally deposited glucocorticoids similarly to the placenta.

### Extraembryonic tissues respond to glucocorticoids even though metabolism is occurring

Research on placental vertebrates has shown that the placenta not only metabolizes glucocorticoids but is also capable of responding to maternal glucocorticoids. This response is mediated through the glucocorticoid receptor, as various isoforms of the glucocorticoid receptor are present in the placenta (Saif et al., 2014). Elevated maternal glucocorticoids can lead to alterations in placental surface area, weight, vascularization, and expression of nutrient transporters (Hahn et al., 1999; Wyrwoll et al., 2009; Fowden et al., 2015). This placental response to glucocorticoids has been shown to result in fetal growth restriction (Clifton et al., 2017; Newnham et al., 1999; Moss et al., 2001). These findings have led to the placenta receiving more attention as a mediator of maternal stress effects that responds to glucocorticoids to regulate embryonic growth. Contrastingly, there is limited research on how avian extraembryonic membranes respond to maternal glucocorticoids, despite evidence these membranes have a critical role in regulating resource availability for growth (Clifton et al., 2017; Noble and Cocchi, 1990, Speake et al., 1998). In chicken eggs, it has been shown that glucocorticoids can reduce angiogenesis in the developing extraembryonic membranes (McNatt et al., 1992), leading to reduced offspring growth (Eriksen et al., 2003; Hayward and Wingfield, 2004). These avian membranes respond to glucocorticoids similarly to the placenta and may be an overlooked mediator of maternal glucocorticoid effects in birds.

The goal of this work was to test whether the extraembryonic membranes of developing chickens (*Gallus gallus*) respond to corticosterone. Previous work identified a gene that was potentially responsive to corticosterone from an unpublished transcriptome obtained using RNAseq on extraembryonic membranes from corticosterone and oil treated eggs. This gene, acyl–CoA thioesterase 13 (*Acot13*) was upregulated in corticosterone treated eggs. *Acot13* is involved in lipid processing and encodes for an enzyme that hydrolyzes fatty acyl-CoAs into free fatty acids and coenzyme A (Powell et al., 2004; Kang et al. 2012). Under physiological conditions, *Acot13* regulates the balance of free fatty acids and the fatty acyl-CoAs that are used for ATP production via β-oxidation in the mitochondria (Kang et al. 2012). Thus, *Acot13* may play an important role in mediating glucocorticoid induced changes in energy dynamics. We subsequently used *Acot13* as a gene that potentially responds to corticosterone to address the following questions: 1) Does a metabolite of corticosterone elicit the same response as corticosterone? 2) What is the duration of the direct response to corticosterone? 3) Does the dose of corticosterone affect the response? This study will provide insight into the mechanisms underlying how maternally deposited glucocorticoids can affect embryonic development.

## RESULTS

### Experiment 1: Response validation, dosage effects, and response duration

**Fig. 1.**
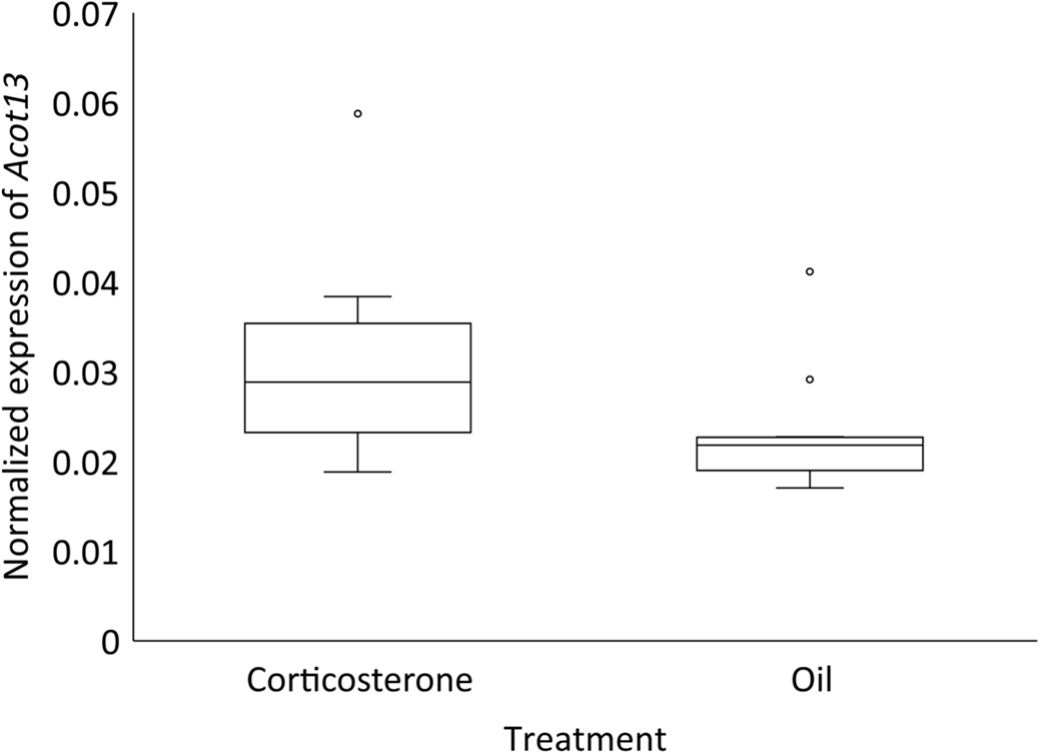
**Normalized *Acot13* expression in the extraembryonic membranes on day four of development in corticosterone and oil treated egg.** *Acot13* expression was significantly elevated (*P*=0.015) in eggs treated with corticosterone (*n*=13) than oil (*n*=13), meaning that the extraembryonic membranes respond to corticosterone. Error bars represent the standard error.

**Fig. 2.**
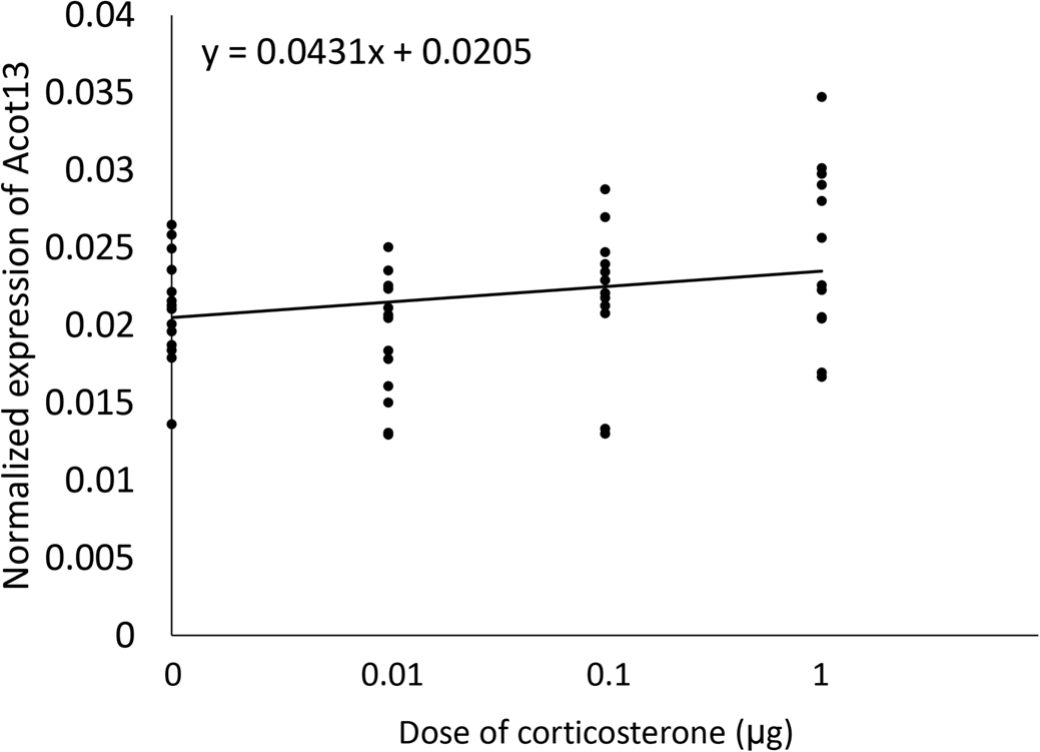
**Normalized *Acot13* expression on day four of development by dose of corticosterone.** There was a significant positive relationship between dose of corticosterone and *Acot13* expression (*P*=0.016). 0 µg, *n*=14; 0.01 µg, *n*=13; 1 µg, *n*=12; 10 µg, *n*=12.

**Fig. 3.**
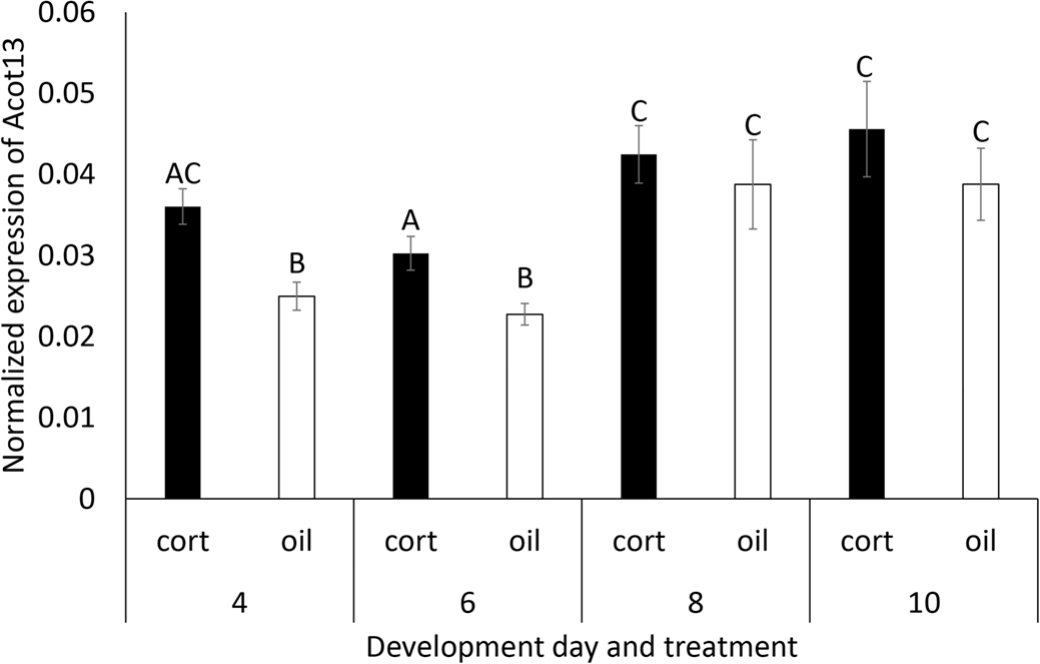
**Normalized *Acot13* expression from days 4 to 10 of development in eggs treated with corticosterone or oil.** Treatment with corticosterone significantly elevated *Acot13* on days four and six, but this effect was transient as expression was not different between groups on days eight or ten. Error bars represent the standard error. *n*=6 for all eight groups.

When validating the response to corticosterone, we found *Acot13* expression was significantly higher in the corticosterone injected eggs than the oil injected eggs on day four of development [*F*(1,23)=6.99, *P*=0.0145] (Fig. 1). Egg mass did not have a significant effect on *Acot13* expression [*F*(1,23)=1.16, *P*=0.2932]. We then tested if the dose of corticosterone affected expression of *Acot13* and found a significant positive relationship between the dose of corticosterone and *Acot13* expression [*F*(1)=6.17, R^2^=0.1118. *P*=0.0165] (Fig. 2). Finally, we characterized the response duration and found a significant main effect of treatment on *Acot13* expression [*F*(3,40)=11.86, *P*=0.0014] and sampling day on *Acot13* expression [*F*(3,40)=10.39, *P* <0.0001] (Fig. 3). A Tukey-Kramer post hoc comparison indicated there was a significant difference in *Acot13* expression between days four and six of development (*P*<0.0001), days four and ten (*P*=0.0022), and days six and ten (*P*<0.0001) (Fig. 3).

### Experiment 2: Testing the effects of corticosterone metabolites on ***Acot13*** expression

**Fig. 4.**
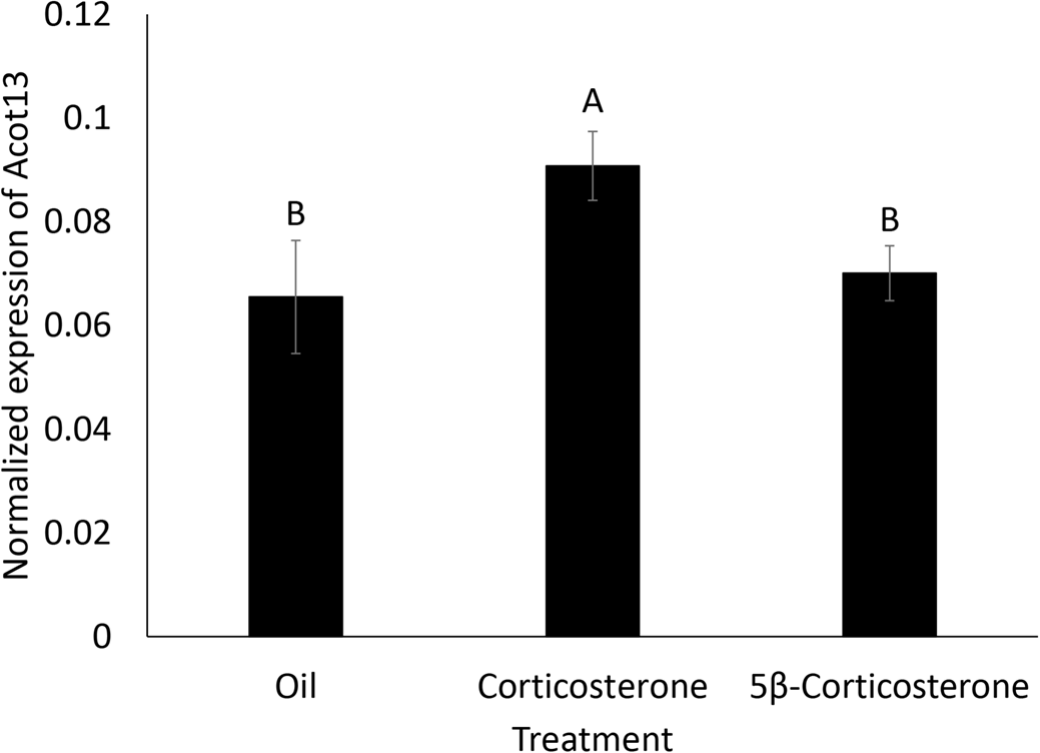
**Normalized *Acot13* expression in the extraembryonic membranes on day four of development of eggs treated with oil, corticosterone, or 5β-corticosterone.** Treatment with corticosterone (*n*=12) significantly elevated *Acot13* expression, but treatment with 5β-corticosterone (*n*=14) did not, meaning metabolism inactivates corticosterone and prevents the extraembryonic membranes from responding. Error bars represent the standard error.

When determining if metabolism of corticosterone inactivates corticosterone, we found there was a significant effect of treatment on *Acot13* expression [*F*(2,35)=4.71, *P*=0.0154] (Fig. 4). *Acot13* expression was significantly higher in the corticosterone injected eggs than the oil injected eggs (*P*=0.003) and the 5β-corticosterone injected eggs (*P*=0.0157) on day four of development. There was no statistical difference between the oil injected eggs and the 5β-corticosterone injected eggs (*P*=0.777).

## DISCUSSION

In this study, we characterized how avian extraembryonic membranes respond to glucocorticoids early in development. We found that elevated glucocorticoids induced *Acot13* expression in the extraembryonic membranes on day four of development (Fig. 1). This response was dose dependent as *Acot13* expression increased as the dose of corticosterone increased (Fig. 2). The response of *Acot13* to glucocorticoids was also transient in that expression was only elevated in corticosterone treated eggs on day four and six of development, but this effect dissipated by day eight and ten (Fig. 3). The timing of the *Acot13* response largely coincides the period of development during which corticosterone is being metabolized, as injected corticosterone is metabolized by day four of development (Harders et al., 2023). We found that the primary metabolite of corticosterone, 5β-corticosterone, did not induce expression of *Acot13* as corticosterone did (Fig. 4), providing further support for the idea that corticosterone metabolites are inactivated. These results give insight into the mechanisms underlying the extraembryonic membrane's response to yolk glucocorticoids early in development.

These results begin to identify when, where, and how yolk glucocorticoids affect development. Numerous studies have demonstrated that yolk glucocorticoids (von Engelhardt et al., 2009; Vassallo et al., 2014, 2019) and other yolk steroids (Paitz et al., 2011, 2020; Campbell et al., 2020; Paitz and Cagney, 2019; Kumar et al., 2019a) are rapidly metabolized during early development. Our results suggest that the extraembryonic membranes may be responding to these steroids before they are metabolized since 5β-corticosterone did not induce *Acot13* expression (Fig. 4). Previous studies have found steroid receptors, such as the progestin, androgen, estrogen, and glucocorticoid receptors are expressed in the extraembryonic membranes of oviparous species early in development (Albergotti et al., 2009; Kumar et al., 2019b). Steroid receptors in the extraembryonic membranes may be responsible for many of the observed effects of yolk steroids (Kumar et al., 2019b). Additionally, because this response occurs in the extraembryonic membranes, the size/functioning of extraembryonic membranes themselves could be altered and, therefore, their ability to transport nutrients from the yolk to the developing embryo. This membrane response does not require maternal glucocorticoids to reach the embryo, allowing maternal effects to occur via changes in membrane function, which could have broad effects on embryonic development through changes in nutrient transport. This extraembryonic response to glucocorticoids has been documented in placental species, as elevated maternal glucocorticoids can reduce the size, vascularization, and amount of glucose transporters present in the placenta, which ultimately affect the embryonic development (Braun et al., 2013, Fowden et al., 2008).

Exposure to corticosterone elevated expression of *Acot13* in the extraembryonic membranes (Fig. 1). *Acot13* is a gene that regulates lipid processing by producing free fatty acids upon cleaving fatty acyl-CoAs into coenzyme-A and free fatty acids (Kang et al., 2012). This metabolism of fatty acyl-CoAs would limit β-oxidation of lipids as only fatty acyl-CoAs can enter the mitochondria and be oxidized for ATP production, ultimately limiting lipid processing and utilization (Tillander et al., 2017). By inducing *Acot13*, corticosterone may mobilize free fatty acids from the extraembryonic membranes that can be used by other embryonic tissues. We found *Acot13* expression was elevated on days four and six of development, but by day eight, *Acot13* expression was not different between the corticosterone and control treatment groups (Fig. 3). Although corticosterone's direct effect on *Acot13* expression may be transient, this response could initiate a cascade of events that persist after *Acot13* expression returned to baseline. Longer term effects could be changes in lipid processing and energy availability, which would affect the embryo, as the major nutrient resources for avian embryos are lipids (Speake et al., 1998). Limiting lipid utilization by the embryo could result in growth restriction, a common phenotype observed due to embryonic exposure to glucocorticoids (Braun et al., 2013, Moss et al., 2001). Beyond altering phenotype, altering lipid processing could potentially be lethal for embryos by causing lipotoxicity due to the accumulation of free fatty acids. Lipotoxicity could be one mechanism of mortality caused by embryonic exposure to glucocorticoids early in development.

Glucocorticoids may be metabolized to protect the embryo from the potentially lethal effects of glucocorticoid exposure. A previous study found that this metabolism was sufficient to prevent embryonic mortality from corticosterone exposure, as exposure to corticosterone metabolites did not induce mortality to the same degree as corticosterone (Harders et al., 2023). Here, we found further evidence that that the corticosterone metabolite, 5β-corticosterone, was inactivated as it did not induce expression of *Acot13* in the extraembryonic membranes compared to corticosterone exposed embryos (Fig. 4). Since yolk corticosterone is metabolized early in development (von Engelhardt et al., 2009; Vassallo et al., 2014, 2019), there may be a limited amount of time that the membranes can respond directly to corticosterone. But what proportion of maternally deposited corticosterone induces *Acot13* expression? We hypothesize the membranes respond to a fraction of maternally deposited corticosterone that escaped metabolism, as the extraembryonic membranes mediate metabolism by releasing the enzyme 5β-reductase into the yolk, which is not homogenous as solid lipids settle to the bottom, forming an aqueous layer near the top. This aqueous barrier would limit the movement of large quantities of lipophilic steroids from the yolk into the membranes. However, some corticosterone that has escaped metabolism may be taken up into the membranes via endocytosis and elicit the effects that we characterized (Vassallo et al., 2019). To note, we only measured one potential pathway that responds to corticosterone, however, there may be other pathways/processes that respond to corticosterone or metabolites, such as angiogenesis (McNatt et al., 1992).

Taken together, these results demonstrate that the extraembryonic membranes respond to yolk glucocorticoids. The early response we characterized could have long lasting effects on embryonic development through changes in lipid processing. We found avian extraembryonic membranes respond to glucocorticoids in a comparable way as the placenta, which would ultimately affect nutrient availability for the offspring. Because the extraembryonic response to glucocorticoids is similar across reproductive methods, there needs to be more emphasis on studying the response to glucocorticoids by extraembryonic membranes in oviparous species. Many questions remain about how the metabolic buffer is regulated, and downstream responses by the extraembryonic membranes to glucocorticoids.

## MATERIALS AND METHODS

### Experiment 1: Response validation, dosage effects, and response duration

#### Response validation

To validate that *Acot13* expression responds to corticosterone manipulations, we first quantified the expression of *Acot13* in the extraembryonic membranes on day four of development. Freshly laid chicken (*G. gallus*) eggs were purchased from the University of Illinois (Urbana, IL, USA) poultry farm and injected with either 1 µg of corticosterone dissolved in 10 µl of vegetable oil (*n*=6) or 10 µl of vegetable oil (*n*=6) as a control. Eggs were incubated at 37°C in 65% humidity for 4 days. All embryo work was carried out in accordance with methods approved by the Illinois State University Institutional Animal and Care Use Committee (IACUC). A portion of the extraembryonic membrane was put in 800 µl of TRIzol and stored at −20°C until RNA extraction. cDNA was synthesized using the Thermo Scientific Maxima First Strand cDNA Synthesis Kit (#K1671). Primers for *Gapdh* (NCBI Reference Sequence: NM_204305.2) and *Acot13* (NCBI Reference Sequence: NM_001407321.1) were developed in the laboratory using the *G. gallus* genome (Warren et al., 2017) and are as follows: *Gapdh* forward GGTCACGCTCCTGGAAGATAGT, *Gapdh* reverse GGGCACTGTCAAGGCTGAGA, *Acot13* forward CCAACAGAGGTGGCACGTTA, and *Acot13* reverse ACCCCAGGCAATGCTCTTT. The amplification factor for each primer pair was determined with a serial dilution of pooled cDNA from extraembryonic membranes and was found to be 1.93 and 1.97 for *Gapdh* and *Acot13*, respectively. RT-qPCR was performed for each sample in triplicate, using PowerUp SYBR Green master mix (Applied Biosystems, Waltham, MA, USA) as the indicator. The thermocycler conditions were initial polymerase activation at 95°C for 20 s, followed by 40 cycles of denaturation at 95°C for 1 s and extension/annealing at 60°C for 20 s. Relative expression to reference gene, *Gapdh*, was calculated using the 2-ΔCT (Schmittgen and Livak, 2008).

#### Dosage effects

To examine how the dose of corticosterone affected *Acot13* expression in the extraembryonic membranes on day four of development, fertile eggs were injected with 1 µg (*n*=12), 0.1 µg (*n*=12), 0.01 µg (*n*=13), or 0 µg (*n*=14) of corticosterone. Since the 1 µg dose was known to induce *Acot13* but is likely on the upper end of the physiological range, we tested a range of lower doses that were within the physiological range. Our doses correspond to concentrations of 17, 1.7, and 0.17 ng/g respectively, based on the average chicken egg weighing ∼60 g. The extraembryonic membranes were sampled on day four and stored in 800 µl of TRIzol. Gene expression was quantified as described above.

#### Response duration

To characterize the duration over which *Acot13* expression is affected by corticosterone, we quantified gene expression in the extraembryonic membranes over the first 10 days of development. Chicken eggs were injected with either corticosterone (1 µg) or vegetable oil as a control on day zero, incubated, and the extraembryonic membranes were sampled on days four, six, eight, and ten of development (*n*=6 for each injection treatment per sampling day). qPCR was performed as described above to determine expression of *Acot13*.

### Experiment 2: Testing the effects of corticosterone metabolites on ***Acot13*** expression

Since numerous yolk steroids such as testosterone (Paitz et al., 2011) and progesterone (Paitz and Cagney, 2019) are metabolized via 5β-reduction, we tested whether 5β-corticosterone was capable of inducing *Acot13* expression in a manner similar to corticosterone. To compare the effect of corticosterone on *Acot13* expression to the effect of 5β-corticosterone, fertile eggs were injected with 1 µg (*n*=12) of corticosterone, 1 µg (*n*=14) of 5β-corticosterone, or just oil (*n*=13). Eggs were incubated at 37°C in 65% humidity for four days and extraembryonic membranes were collected to quantify *Acot13* expression as described above.

### Statistical analyses

All analyses were run using SAS statistical software (v. 9.4, SAS Institute, Cary, NC, USA). A two-sample *t*-test was used to examine the difference in expression of *Acot13* in the extraembryonic membranes based on treatment. To determine the duration of the response to corticosterone between days four and ten of development, a two-way ANOVA was performed with injection treatment and sampling day as fixed effects. To determine if *Acot13* expression was dependent on the dose of corticosterone, a linear regression was performed. Finally, to determine if a metabolite of corticosterone induced expression of *Acot13*, an ANOVA was performed with injection treatment as a fixed effect. In all the gene expression studies, the data are log10 transformed for analysis. All data can be found in supplementary material.

## Supplementary Material

10.1242/biolopen.060131_sup1 Supplementary information

Table S1.
