# Supplementary material for: Avian extraembryonic membranes respond to yolk corticosterone early in development
Source: Biol Open. 2024 Jan 22;13(1):bio060131. doi: 10.1242/bio.060131 (PMC10836647; doi:10.1242/bio.060131)
Supplement: Supplementary information [file biolopen-13-060131-s1.pdf]

Table S1.

Available for download at
https://journals.biologists.com/bio/article-lookup/doi/10.1242/bio.060131#supplementary-data

Biology Open • Supplementary information
